# Aβ oligomers promote oligodendrocyte differentiation and maturation via integrin β1 and Fyn kinase signaling

**DOI:** 10.1038/s41419-019-1636-8

**Published:** 2019-06-06

**Authors:** Tania Quintela-López, Carolina Ortiz-Sanz, Mari Paz Serrano-Regal, Adhara Gaminde-Blasco, Jorge Valero, Jimena Baleriola, Maria Victoria Sánchez-Gómez, Carlos Matute, Elena Alberdi

**Affiliations:** 1 Department of Neuroscience, University of Basque Country (UPV/EHU), Leioa, 48940 Spain; 2 Achucarro Basque Center for Neuroscience, Leioa, 48940 Spain; 3 CIBERNED, Leioa, 48940 Spain; 4 IKERBASQUE Basque Foundation for Science, Bilbao, Spain; 5 Department of Cell Biology and Histology, UPV/EHU, Leioa, 48940 Spain

**Keywords:** Cellular neuroscience, Oligodendrocyte

## Abstract

Alzheimer´s disease (AD) is characterized by a progressive cognitive decline that correlates with the levels of amyloid β-peptide (Aβ) oligomers. Strong evidences connect changes of oligodendrocyte function with the onset of neurodegeneration in AD. However, the mechanisms controlling oligodendrocyte responses to Aβ are still elusive. Here, we tested the role of Aβ in oligodendrocyte differentiation, maturation, and survival in isolated oligodendrocytes and in organotypic cerebellar slices. We found that Aβ peptides specifically induced local translation of 18.5-kDa myelin basic protein (MBP) isoform in distal cell processes concomitant with an increase of process complexity of MBP-expressing oligodendrocytes. Aβ oligomers required integrin β1 receptor, Src-family kinase Fyn and Ca^2+^/CaMKII as effectors to modulate MBP protein expression. The pharmacological inhibition of Fyn kinase also attenuated oligodendrocyte differentiation and survival induced by Aβ oligomers. Similarly, using ex vivo organotypic cerebellar slices Aβ promoted MBP upregulation through Fyn kinase, and modulated oligodendrocyte population dynamics by inducing cell proliferation and differentiation. Importantly, application of Aβ to cerebellar organotypic slices enhanced remyelination and oligodendrocyte lineage recovery in lysolecithin (LPC)-induced demyelination. These data reveal an important role of Aβ in oligodendrocyte lineage function and maturation, which may be relevant to AD pathogenesis.

## Introduction

Alzheimer’s disease (AD) is characterized by a progressive increase in the amount of soluble and insoluble amyloid-β (Aβ) in various molecular forms such as monomers, oligomers, insoluble fibrils, and plaques in the central nervous system (CNS). New lines of evidences support the concept that an imbalance between Aβ production and clearance of Aβ peptides is a very early, often initiating factor in AD^1,2^. Aβ oligomer accumulation impairs learning and memory and instigate major facets of AD neuropathology, including tau hyperphosphorylation, synapse deterioration and loss, inflammation, and oxidative damage^3^. In addition to gray matter pathology, white matter (WM) changes are now recognized as an important pathological feature in the emergence of the disease. Levels of Aβ oligomers are higher in WM of AD patients compared with non-AD controls^4^. In fact, in preclinical stages, cerebrospinal fluid (CSF) biomarkers for amyloid pathology are closely associated with myelin alteration, which suggests that these abnormalities may signify an early feature of the disease process^5^. Several studies proposed that Aβ alters myelinating oligodendrocyte functions and induces toxicity^6–9^, although mechanisms involved are still poorly characterized.

Oligodendrocytes are the myelin-producing cells of the CNS, and wrap layers of lipid-dense insulating myelin around axons. Proteolipid protein (PLP) and myelin basic protein (MBP) are the most abundant proteins in the CNS myelin and whereas knockout mouse for PLP does not show myelination deficits^10^, a lack of MBP results in failure to form compact myelin leading to shivering symptoms and premature death^11^. MBP contributes to the compaction of myelin membranes^12^ and as well as in cytoskeletal assembly, membrane extension, and maintenance of Ca^2+^ homeostasis^13^. Fyn kinase is a crucial signaling molecule that converts axonal signals into local translation of MBP^14^ and participates in oligodendrocyte differentiation and myelination^15,16^. In addition, Fyn is a key protein in signaling mechanisms of Aβ peptides in neurons^17^. Therefore, identifying the effects of Aβ peptides in Fyn-dependent pathways in oligodendrocyte cell lineage will provide useful information for understanding the contribution of these cells to AD pathology.

Here, we analyzed the changes on MBP levels and on dynamics of oligodendroglia lineage population lead by Aβ oligomers. Our results indicate that oligomeric forms increase MBP translation in oligodendrocyte processes, and promote morphological differentiation, as well as cell survival in cultured oligodendrocytes by mechanisms involving integrin β1 (Itgb1) signaling, Fyn kinase and intracellular Ca^2+^ modification. Moreover, Aβ contributes to oligodendrocyte differentiation in cultured cerebellar slices and enhances remyelination and oligodendrocyte lineage recovery in lysolecithin (LPC)-induced demyelination.

## Results

### Aβ oligomers induce local synthesis of MBP by promoting MBP messenger RNA (mRNA) translation in distal processes of oligodendrocytes

**Fig. 1:**
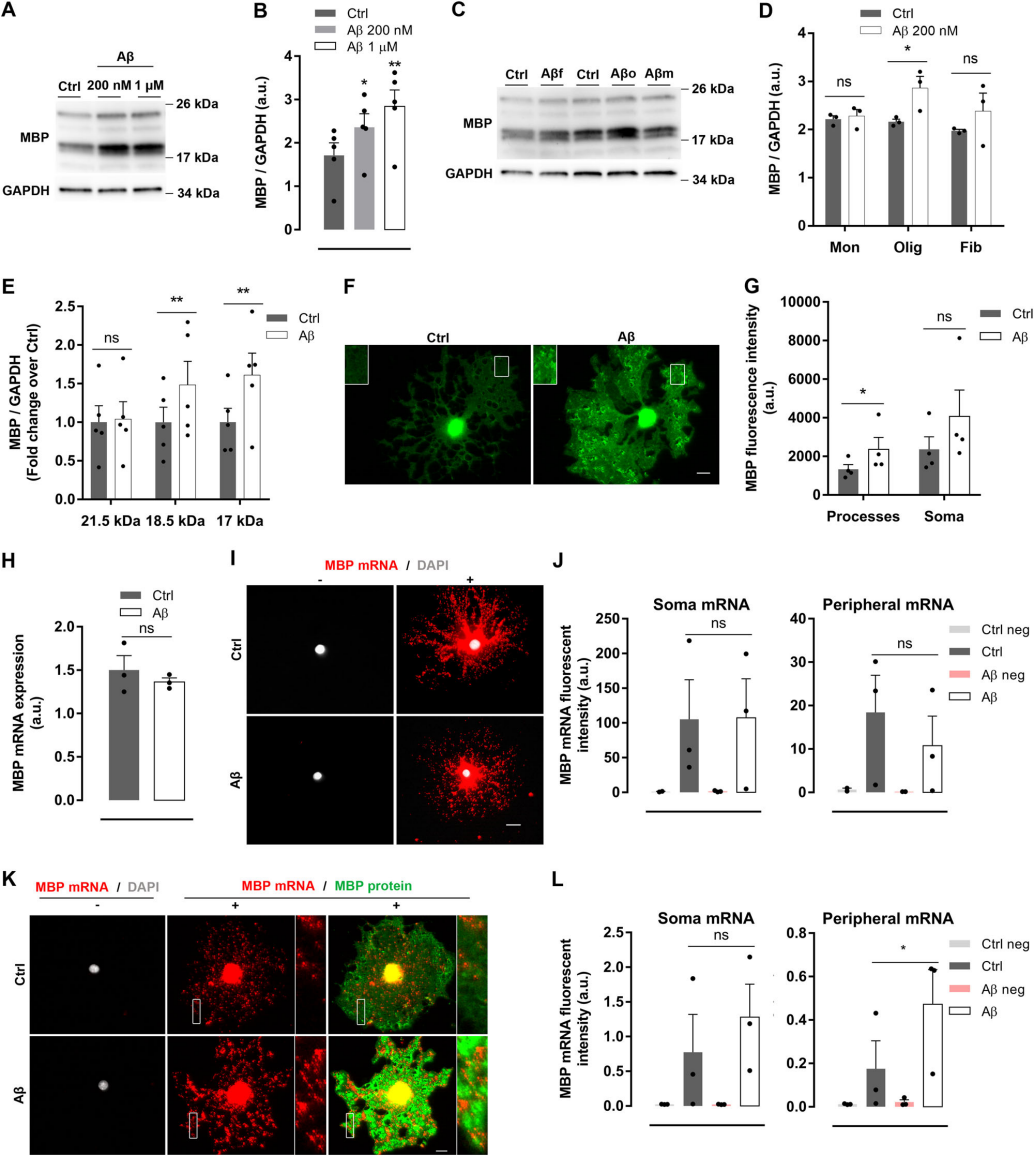
Aβ oligomers induce local synthesis of MBP by promoting MBP mRNA translation in distal processes in cultured oligodendrocytes. **a**, **b** MBP expression and relative quantification in total cell extracts from oligodendrocytes treated with Aβ 200 nM or 1 µM for 24 h was analyzed by western blotting (*n* = 5). **c**, **d** Cells were treated with fibrillar, monomeric or oligomeric Aβ peptide (Aβf, Aβm, and Aβo, respectively) at 1 µM for 24 h, and total MBP levels in whole extracts were detected and quantified by western blot (*n* = 3). **e** Western blotting analysis of the expression of MBP isoforms (21.5 kDa, 18.5 kDa, and 17 kDa) in oligodendrocytes treated with Aβ 1 µM for 24 h (*n* = 5). **f**, **g** MBP-immunolabeled micrographs and analysis of MBP fluorescence intensity of 1 µM Aβ-treated (Aβ) or non-treated (Ctrl) oligodendrocytes. Scale bar, 10 µm (*n* = 4). **h–l** MBP mRNA levels were measured after Aβ 1 µM exposure for 24 h by RT-qPCR (**g**) and quantitative FISH in the presence (**i**, **j**) or absence (**k**, **l**) of protease. **i**, **k** Representative epifluorescence images of cultured oligodendrocytes showing MBP mRNA (red) non-targeting probe (-), positive probe (+), and MBP protein detected by immunolabeling (green). **j**, **l** Quantitative FISH analysis of MBP mRNA fluorescence intensity of soma and distal processes (*n* = 3). Scale bar, 10 µm. Data are represented as means ± S.E.M and were analyzed by paired Student´s *t-*test (**a**, **g–l**) and one-way ANOVA followed by Holm-Sidak’s multiple comparisons test (**b**). *p < 0.05, **p < 0.01 compared to non-treated cells

To assess the impact of Aβ oligomers on cultured oligodendrocytes, we analyzed MBP, PLP, MAG (myelin-associated glycoprotein), and enzyme CNPase levels. First, we observed that levels of MBP protein increased in oligomeric Aβ-treated cells compared to controls (Fig. 1a, b and Supplementary Fig. 1), while the other myelin proteins were unchanged (Supplementary Fig. 2a, b). Moreover, MBP upregulation was specific for Aβ oligomeric forms since no changes were found in cells treated with fibrillar and monomeric Aβ species^18,19^ (Fig. 1c, d). Isoforms of MBP range from 14 to 21.5 kDa and play different roles during oligodendrocyte development^13^. Here, we observed that oligomeric Aβ specifically modulates the expression of 17–18.5 kDa isoforms but not 21 kDa MBP isoform. Additionally, inmunocytochemistry analysis of MBP distribution showed that Aβ increased MBP fluorescence intensity only at distal processes, while no differences were found at soma area (Fig. 1e–g). Interestingly, higher levels of MBP were observed in hippocampal samples of 6, 12, and 18-month-old 3xTg-AD mice (Supplementary Fig. 3a, b), indicating that synthesis of this protein is dysregulated in adult and aging mice overexpressing Aβ oligomers. Overall, these results indicate that oligomeric forms of Aβ peptide upregulate oligodendrocyte MBP expression and its subcellular distribution at distal processes.

Next, we asked whether induction of MBP levels in response to Aβ occurred at a transcriptional and/or translational level. First, we measured total MBP mRNA by quantitative reverse transcription PCR (RT-qPCR) and no significant differences were found between untreated and Aβ-treated cells (Fig. 1h). These results were corroborated by using quantitative fluorescent in situ hybridization (FISH) using protease treatment to unmask all mRNA molecules and enhance its detection. We found that Aβ did not change MBP mRNA fluorescence intensity in soma and distal processes as compared to non-treated cells (Fig. 1i, j). However, quantification of MBP mRNA levels by FISH in the absence of protease showed that Aβ promoted an increase in the amount of unmasked MBP mRNA in oligodendrocyte distal processes compared to non-treated cells, while no differences were found in soma (Fig. 1k, l). In summary, these results show that oligomeric Aβ increased MBP mRNA local translation in cultured oligodendrocytes.

### Aβ oligomers increase morphological differentiation and maturation in oligodendrocytes

**Fig. 2:**
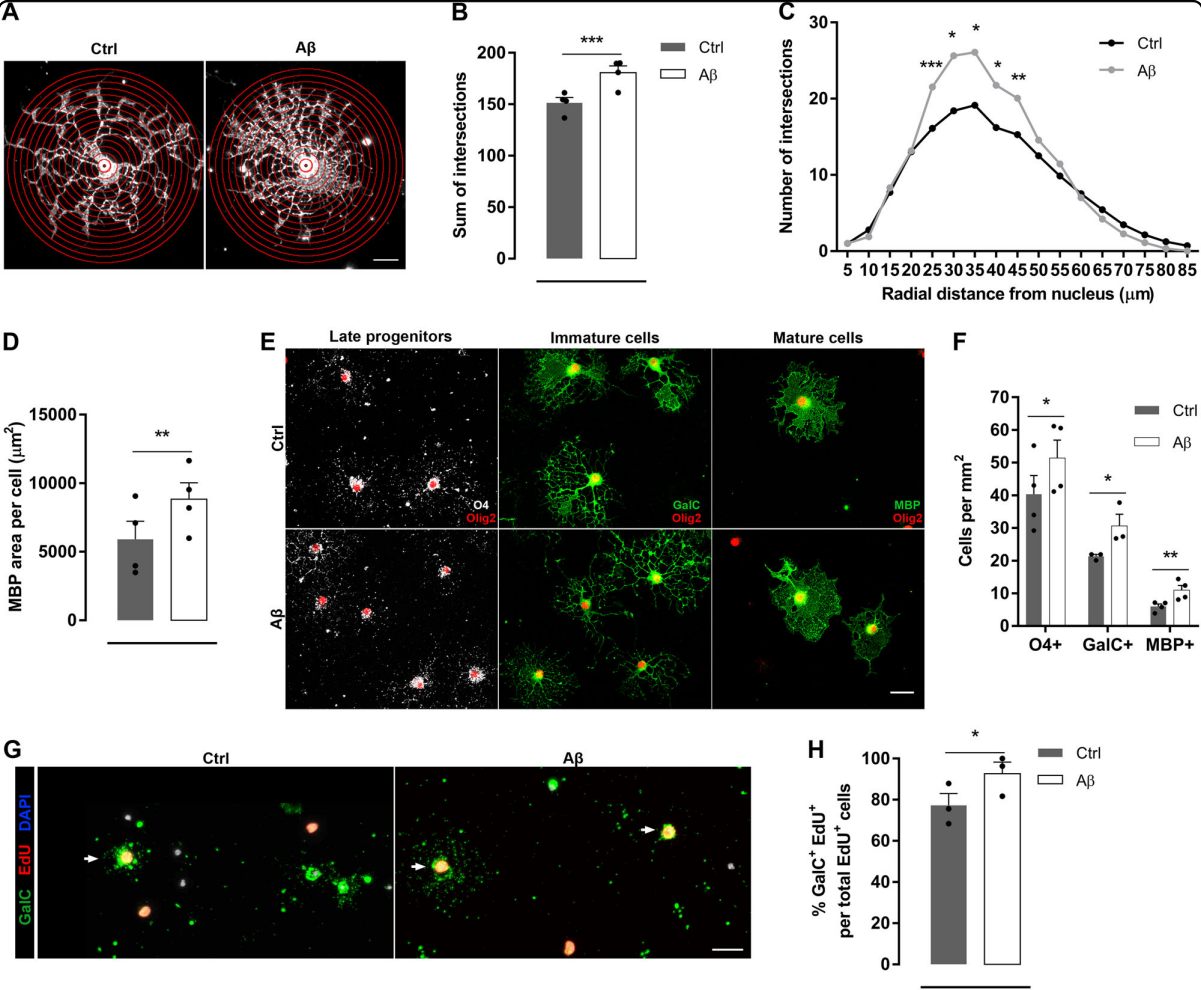
Aβ oligomers increase morphological differentiation and maturation in oligodendrocytes. **a–f** Cells were stimulated in the presence or absence of Aβ 1 µM for 24 h. **a** MBP-labeled non-treated (Ctrl) and Aβ-treated (Aβ) oligodendrocytes with superimposed concentric circles at 5 µm intervals emerging from nucleus. Scale bar, 10 µm **b**, **c** Sholl analysis shows the total number of intersections (**b**) and grouped by the distance from nucleus (**c**). **d** Analysis of the occupied area by the MBP staining per cell (*n* = 4). **e** Representative micrographs showing double-immunostaining for oligodendrocyte linage marker Olig2 (red) and O4 (white, late progenitors), GalC (green, immature oligodendrocytes), or MBP (green, mature oligodendrocytes). **f** The number of specific positive cells was counted after treatment (*n* = 3–4 cultures). **g** Double immunolabelling of GalC (green) and EdU (red) in Ctrl and Aβ-treated oligodendrocytes. Arrow indicates new inmature oligodendrocyte (GalC^+^EdU^+^). Cell nuclei was visualized by DAPI (blue). **h** Quantification of the number of newly generated mature oligodendrocytes (GalC^+^EdU^+^) (*n* = 3) (**g**). Scale bar, 25 µm. Data are represented as means ± S.E.M and were analyzed by paired Student´s *t*-test. *p < 0.05, **p < 0.01, ***p < 0.001 compared to non-treated cells

To investigate the role of Aβ oligomers in oligodendrocyte morphological differentiation and maturation, we quantified process complexity by Sholl analysis of MBP^+^ cells (Fig. 2a). Quantitative analysis detected a significant increase in the number of Sholl ring crossings indicative of increased process branching and extensions from the cell body in Aβ-treated cells compared to control non-treated cells (Fig. 2b). Differences in cell morphology were more prominent in the Aβ-treated oligodendrocyte processes distant 25–45 µm from nucleus (Fig. 2c). Additionally, Aβ promoted an increase in the cellular area occupied by MBP (Fig. 2d), indicating a more mature morphological state of cells.

Typically, the differentiation state of oligodendroglia is determined by lineage-specific cell markers. Olig2 is maintained throughout oligodendroglial lineage, while O4 expression begins in late progenitors^20^. Inmature and mature oligodendrocytes express GalC and MBP, respectively. In order to evaluate the differentiation state of cells after Aβ exposure, we performed immunocytochemistry for the above stage‐specific cell antigens. Number of O4^+^, GalC^+^, and MBP^+^ cells increased significantly compared to non‐treated cells (Fig. 2e, f). Additionally, the proportion of newly generated oligodendrocytes GalC^+^EdU^+^ was increased after Aβ exposure (Fig. 2g, h), further demonstrating that Aβ may promote maturation in oligodendroglia lineage. Overall, these results indicated that Aβ oligomers promoted oligodendrocyte differentiation and maturation.

### Aβ oligomers trigger an integrin β1 signaling pathway leading from Src-family kinase Fyn to upregulate MBP protein expression in oligodendrocytes

**Fig. 3:**
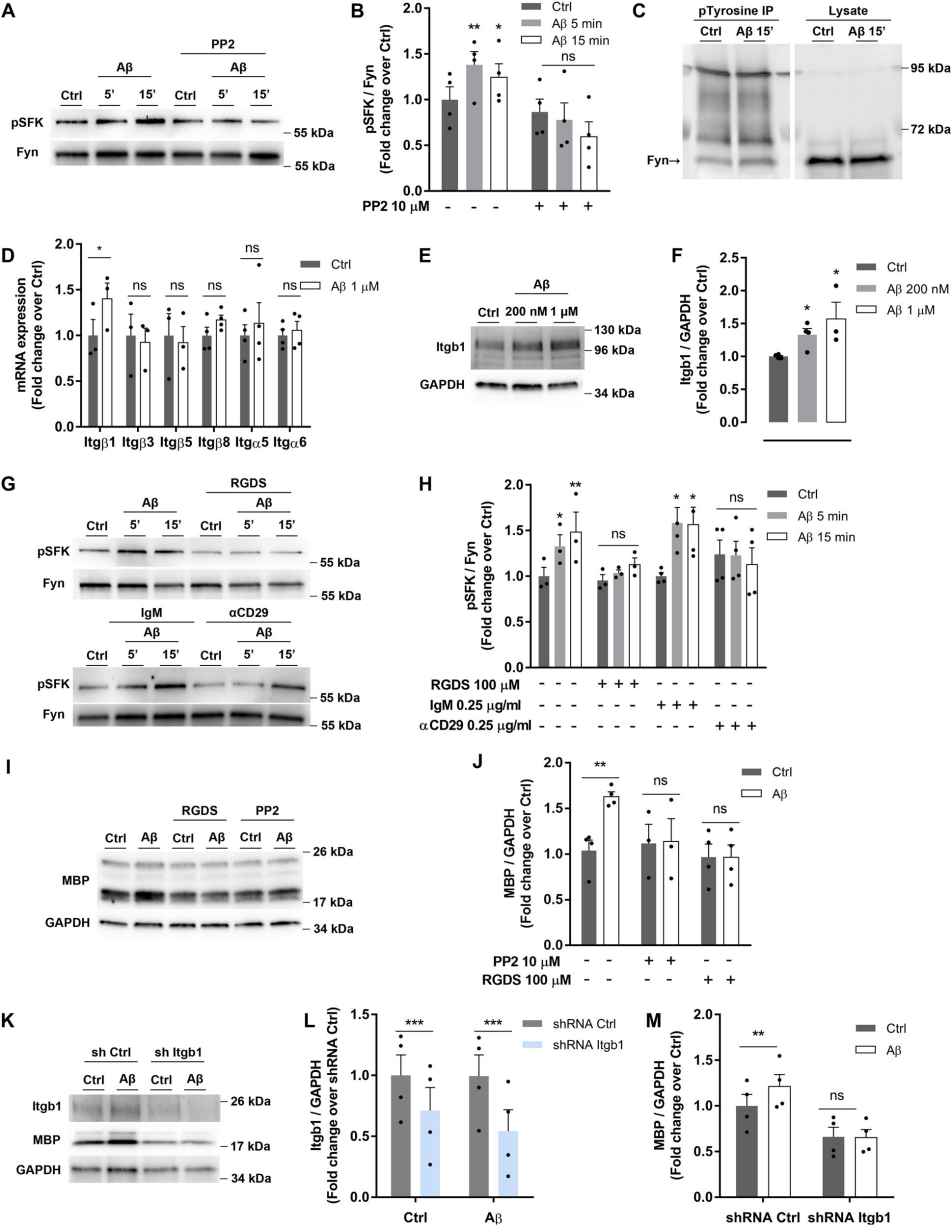
Integrin β1 mediates Aβ-induced MBP upregulation through Fyn activation in oligodendrocytes. **a**, **b** Cells were exposed to Aβ 200 nM for 5 or 15 min, in the presence or absence of PP2, a SFK inhibitor, and phosphorylation of Src-family kinases was examined (pSFK) by western blot (*n* = 4). **c** Immunoprecipitation (IP) of proteins from cells treated with Aβ 200 nM for 15 min was performed with phosphotyrosine (ptyrosine) followed by Fyn detection by western blot. Total cell lysates were immunoblotted with Fyn. **d** Integrin receptor β (Itgβ) and α (Itgα) subunit expression levels in cells treated with Aβ (200 nM, 24 h) were analyzed by RT-qPCR (*n* = 3–4). **e**, **f** Western blot analysis of Itbβ1 receptor in cells treated with Aβ 200 nM or 1 µM for 24 h (*n* = 4). **g**, **h** Cells were treated with Aβ 200 nM for 5 and 15 min after preincubation with RGDS (integrin inhibitor) or CD29 antibody (specific integrin β1 inhibitor) and pSFK levels normalized to Fyn were detected by western blot. (*n* = 4). Non-immune IgM was used as control. **i**, **j** MBP expression in cells treated with Aβ 200 nM for 24 h and preincubated with RGDS or PP2 was analyzed by western blot (*n* = 4). **k**, **l**, **m** Lentiviral transduction of oligodendrocytes with control shRNA (sh ctrl) or shRNA targeting Itgb1 (sh Itgb1) for 24 h. Cells were treated with Aβ for 24 h, and Itbβ1 (**l**) and MBP (**m**) protein levels were measured by western blot. Data are represented as means ± S.E.M and were analyzed by two-way ANOVA followed by Bonferroni posttest (**b**, **h**), one-way ANOVA followed by Bonferroni posttest (**f**) and paired Student´s *t*-test (**j**, **l**, **m**). *p < 0.05, **p < 0.01, ***p < 0.001 compared to non-treated cells or sh ctrl

To determine the molecular pathway underlying Aβ-induced MBP upregulation, we focused on phosphorylated kinase Fyn at tyrosine 418 since it is required for its full catalytic activity and for initial events of axonal myelination^14^. Oligomeric Aβ treatment for 5 and 15 min increased phosphorylation of Src-family kinases (pSFK) in oligodendrocytes, an effect that was blocked in presence of PP2, an specific inhibitor of Fyn (Fig. 3a, b). Immunoprecipitation assays with anti-phosphotyrosine antibody and western blot detection using Fyn and Src antibodies showed that Fyn was the specific member of SFKs activated by Aβ, being the one phosphorylated upon Aβ exposure (Fig. 3c). In contrast, Aβ did not promote tyrosine phosphorylation of Src (Supplementary Fig. 4a), and Lck expression was not detected in cultured oligodendrocytes (Supplementary Fig. 4b), both proteins being members of SFK.

Next, the involvement of a receptor to mediate Fyn activation by Aβ oligomers was studied. Integrin β1 (Itgb1) increases Fyn activity^21^ and enhances MBP expression of oligodendrocytes^22^. First, we characterized expression of integrin subunits by RT‐qPCR (Fig. 3d) and western blot (Fig. 3e, f) in isolated oligodendrocytes. Aβ induced Itgb1 mRNA and protein overexpression, as previously described in astrocytes^23^. Next, western blot analysis showed that Aβ-induced Fyn phosphorylation was attenuated by preincubation with RGDS peptide, which contains the integrin binding sequence and inhibits its function^24^. Similarly, treatment with antibody αCD29 (that specifically binds Itgb1 and blocks its activity)^25^ reduced Fyn phosphorylation, which was not observed with the isotype control (Fig. 3g, h). Consequently, RGDS peptide and PP2 inhibitor blocked the rise of MBP levels induced by Aβ for 24 h (Fig. 3i, j), indicating that Fyn and integrin receptors were involved in Aβ‐modulated MBP expression. Additionally, Itgb1 shRNA lentiviral particles reduced the target gene expression when compared to cells treated with control shRNA (Fig. 3k, l). Thus, cells with silenced Itgb1 and treated with Aβ did not exhibit increased MBP levels compared to non-silenced cells (Fig. 3k, m). These results pointed to Itgb1 as a receptor for Aβ oligomers in oligodendrocytes mediating Aβ-induced Fyn phosphorylation and MBP overexpression.

### Calcium and CaMKII are required for Aβ-induced Itgb1/Fyn signaling to drive oligodendroyte MBP changes

**Fig. 4:**
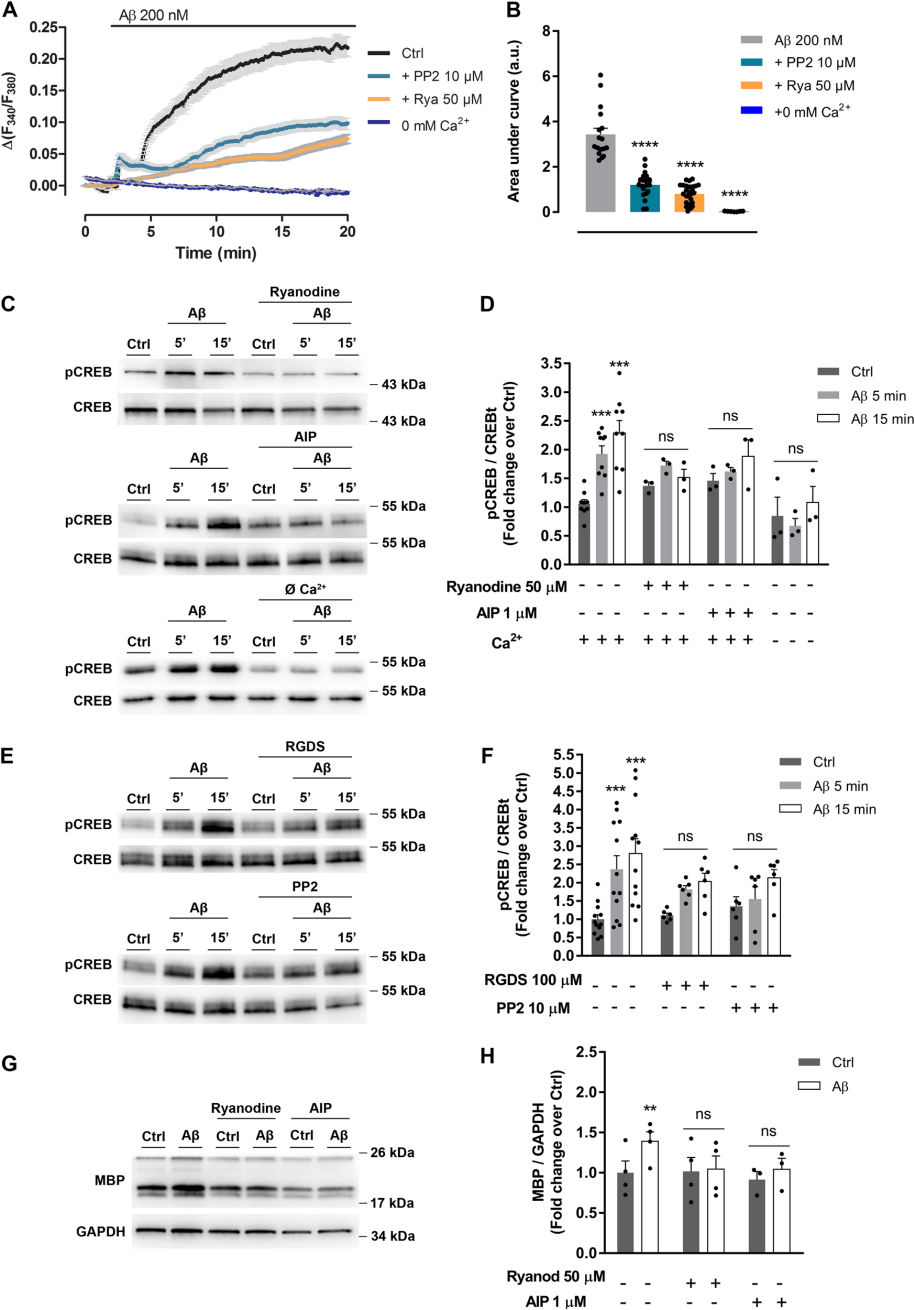
Aβ-induced intracellular Ca^2+^ increase and CaMKII activation upregulate MBP expression in oligodendrocytes. **a** Cells were exposed to Aβ oligomers and Ca^2+^ levels were measured in Ca^2+^-containing extracellular buffer in the presence of PP2 (SFK inhibitor) or the RyR inhibitor ryanodine, and in Ca^2+^-free extracellular buffer (*n* = 4). **b** Bars represent the mean ± SEM of Ca^2+^ increases, quantified as the area under the curve in treated oligodendrocytes. **c**, **d** Western blot of CREB phosphorylation (pCREB) in cells treated with Aβ 200 nM for 5 or 15 min, and preincubated with ryanodine or AIP (CaMKII inhibitor). pCREB levels were also analyzed in the presence or absence of Ca^2+^ (*n* = 3; lower panel). **e**, **f** Cells were preincubated with RGDS (integrin inhibitor) or PP2 (SFK inhibitor) and treated with Aβ 200 nM for 5 or 15 min. CREB activation was detected and analyzed by western blot (*n* = 6). **g**, **h** Cells with ryanodine or AIP, were treated with Aβ 200 nM for 24 h and MBP expression levels were examined by western blot. Data are represented as means ± S.E.M and were analyzed by one-way ANOVA followed by Holm-Sidak’s multiple comparisons test (**a**, **b**) and two-way ANOVA followed by Bonferroni posttest (**d**, **f**, **h**). *p < 0.05, **p < 0.01, ***p < 0.001 compared to non-treated cells

Ca^2+^ level increases stimulate synthesis of MBP in oligodendrocytes^26^ by mechanisms that may involve CaMKII activities^27^. Recordings of intracellular Ca^2+^ in oligodendrocytes showed a sustained rise of its levels in response to Aβ oligomers, which was blocked by PP2, by ryanodine, a blocker of ryanodine receptors, and by absence of extracellular Ca^2+^ (Fig. 4a, b). To analyze the Aβ-induced activation of CaMKII, we measured phosphorylation of CREB as readout of CaMKII activity. CREB was strongly phosphorylated by Aβ oligomers and Aβ‐induced CREB activation was significantly attenuated by ryanodine, by a CaMKII blocker AIP peptide, by Ca^2+^ free extracellular medium (Fig. 4c, d), and by RGDS peptide and PP2 (Fig. 4e, f). Furthermore, enhancement of MBP expression was totally blocked by ryanodine and AIP (Fig. 4g, h). These results indicated that Aβ oligomers promoted changes on intracellular Ca^2+^ levels and CaMKII activity, which are Itgb1/Fyn-dependent. Moreover, Ca^2+^ and CaMKII activity were required for Aβ-induced MBP overexpression.

### Aβ oligomers enhance oligodendrocyte survival in vitro

**Fig. 5:**
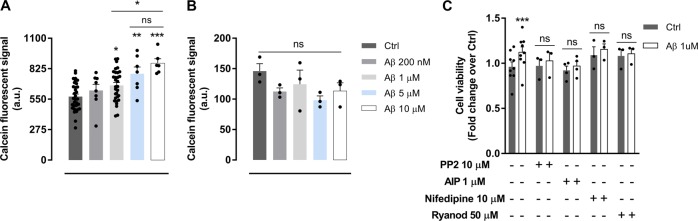
Stimulation of oligodendrocytes with Aβ oligomers enhances cell survival in vitro. **a** Cells were treated with increasing concentrations of Aβ (200 nM–10 µM) for 24 h and viability was quantified by vital dye calcein-AM assay (*n* = 6–20). **b** Cell culture medium without cells in the presence or absence of Aβ was incubated for 24 h and chemical interaction between Aβ and calcein-AM was analyzed (*n* = 3). **c** Oligodendrocyte viability was measured by calcein-AM method after incubation with Aβ 1 µM alone or combined with intracellular inhibitors, PP2 (SFK inhibitor), AIP (CaMKII inhibitor), nifedipine (Ca^2+^channel inhibitor), or ryanodine (ryanodine receptor blocker) (*n* = 3–4). Data are represented as means ± S.E.M and were analyzed by one-way ANOVA followed by Holm-Sidak’s multiple comparisons test (**a**, **b**) and two-way ANOVA followed by Bonferroni posttest (**c**). *p < 0.05, **p < 0.01, ***p < 0.001 compared to non-treated cells

Integrin ligation and activation by extracellular matrix proteins appears to be a key factor in the survival of oligodendrocytes^28^. To examine the role of Aβ oligomers in oligodendrocyte survival, cells were maintained with Aβ in cultured media for 24 h and cell viability was determined by using the fluorescent vital staining calcein‐AM. Data showed a dose-dependent significant increase in oligodendrocyte survival, reaching a protection plateau for Aβ at 5 μM (Fig. 5a). In addition, to rule out non-specific signals due to an interaction between calcein‐AM probe and Aβ, we added Aβ oligomers to culture media for 24 h in absence of cells and calcein fluorescence intensity was measured. No differences were found between control and Aβ‐conditioned media (Fig. 5b). Then, we investigated the molecular mechanisms underlying Aβ‐enhanced oligodendrocyte survival. Cells were treated with Aβ at 1 μM for 24 h and together with PP2, AIP, ryanodine, or nifedipine (Ca^2+‐^channel blocker). Aβ‐promoted cell viability was blocked by using inhibitors, indicating that Fyn activation and intracellular Ca^2+^ homeostasis are important to oligodendrocyte survival (Fig. 5c).

### Aβ oligomers modulate Fyn-dependent MBP overexpression and oligodendrocyte differentiation in organotypic slices

**Fig. 6:**
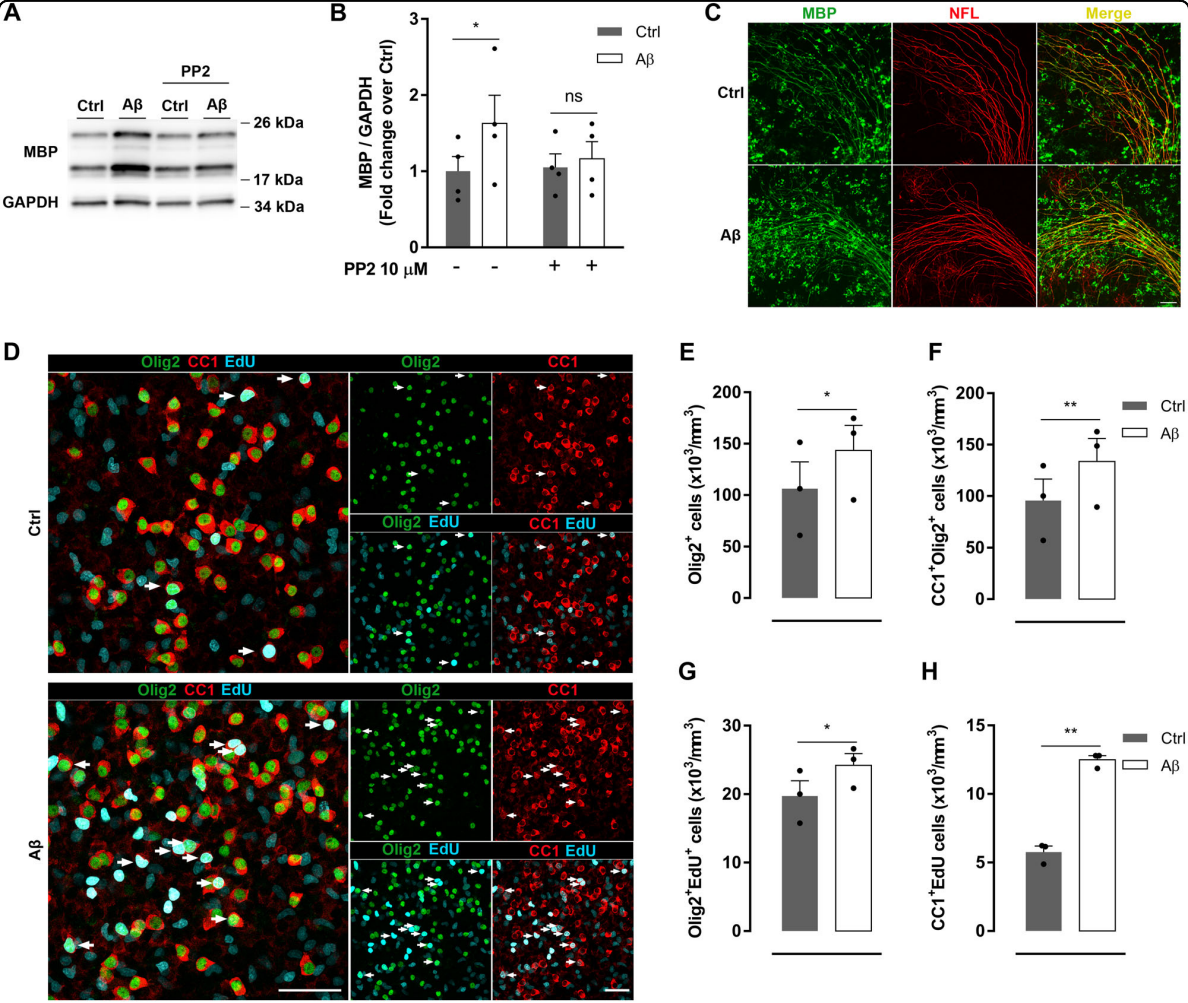
Aβ oligomers promote oligodendrocyte proliferation and differentiation in cerebellar slices. **a**, **b** Cerebellar organotypic slices were treated with Aβ 200 nM for 48 h in the presence or absence of PP2 (SFK inhibitor, 10 µM). MBP levels were analyzed by western blot (*n* = 4). **c** Representative immunofluorescence micrographs of cerebellar slices treated with or without Aβ and stained for neurofilament L (NFL, red), myelin marker (MBP, green), and colocalization of these markers is shown in merged images (yellow). Scale bar, 50 µm. **d–h** Quantification by immunofluorescence of total number of oligodendrocytes (Olig2^+^, green) (**e**) and mature oligodendrocytes (CC1^+^, red) (**f**) in cerebellar slices. Numbers of newly generated Olig2^+^ and CC1^+^ were determined by co-labeling with EdU (cyan) (*n* = 3). Arrows indicate new mature oligodendrocytes (CC1^+^Olig2^+^EdU^+^). Scale bar, 50 µm. Data are represented as means ± S.E.M and were analyzed by two-way ANOVA followed by Bonferroni posttest (**b**) and paired Student´s *t*-test (**e–h**). *p < 0.05, **p < 0.01, compared to non-treated slices

Cerebellar organotypic culture is the most useful model to reproduce all stages of axon myelination^29^, preserving the neuronal networks and neuro-glial interaction. Here, to assess the effects of Aβ in myelination, we treated rat cerebellar slices (7DIV) with Aβ for 48 h. Expression analysis of MBP by western blot and immunofluorescence showed a significant protein overexpression in Aβ-treated slices. (Fig. 6a, b). Moreover, an increase in MBP labeling levels was associated with neurofilament-L (NFL), used as an axonal marker (Fig. 6c), suggesting that a correct myelination was occurring. Consistent with the observations in cultured oligodendrocytes, MBP upregulation induced by Aβ in organotypic slices was mediated by Fyn kinase, since PP2 inhibitor blocked MBP overexpression seen after Aβ treatment (Fig. 6a, b).

Next, we asked whether oligomeric Aβ regulates oligodendrocyte lineage cell development. To address that, the number of total oligodendrocyte lineage cells (Olig2^+^) and mature oligodendrocytes (CC1^+^Olig2^+^) was analyzed. We observed that slices treated with Aβ showed a significant increase in Olig2^+^ and CC1^+^Olig2^+^ cells (Fig. 6d–f). To analyze oligodendrocyte proliferation and differentiation, cells were traced with EdU, a quantitative way to monitor proliferation. Analysis showed that Aβ slightly increased oligodendrocyte proliferation (Olig2^+^EdU^+^), while inducing a strong oligodendrocyte differentiation (CC1^+^EdU^+^) in comparison with control slices (Fig. 6d, g, h). These results indicate that Aβ promoted changes on oligodendrocyte proliferation and differentiation rates in cerebellar organotypic slices.

### Aβ oligomers enhance remyelination and oligodendrocyte lineage recovery in lysolecithin-induced demyelination in organotypic slices

**Fig. 7:**
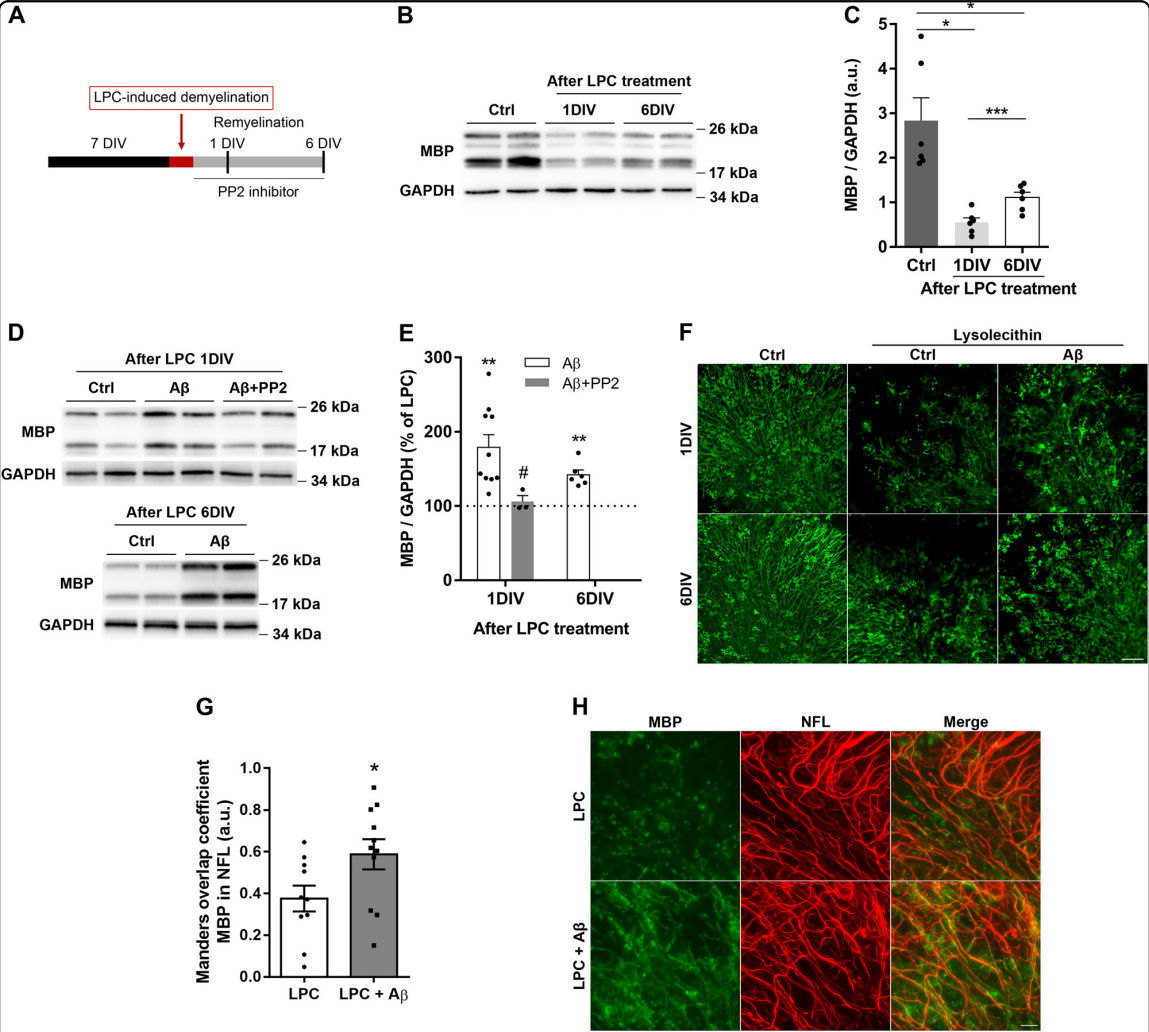
Aβ oligomers enhance remyelination in lysolecithin-treated organotypic slices. **a** Schematic diagram of experimental paradigm. **b**, **c** Cerebellar slices were treated with lysolecithin (LPC) for 16 h and total protein samples were extracted 1 (1DIV) and 6 (6DIV) days after LPC treatment and MBP expression was analyzed by western blot (*n* = 6). **d**, **e** Western blotting of MBP levels was analyzed 1 and 6 days after LPC treatment in the presence or absence of Aβ (200 nM) and PP2 (SFK inhibitor) (*n* = 3–10). **f** Representative images of MBP levels from cerebellar slices treated with LPC and Aβ at two different time points. Scale bar, 50 µm. **g**, **h** Organotypic cerebellar slices at 6 days post-LPC induced demyelination were treated with vehicle, control, or Aβ oligomers during remyelination and immunostained against MBP and axonal neurofilament-H (NFL). **g** Graph bar represents the fiber remyelination index expressed as Manders overlap coefficient of MBP over NFL staining of analysis of 10–11 areas of *n* = 3 experiments. **h** Representative images of MBP and NFL immunostainig are shown. Scale bar 500 µm. Data are represented as means ± S.E.M and were analyzed by one-way ANOVA followed by Holm-Sidak’s multiple comparisons test. *p < 0.05, **p < 0.01, ***p < 0.001 compared to non-treated slices. #*p* < 0.05 compared to Aβ alone (**c**, **e**) and by unpaired Student´s *t-*test *p < 0.05 compared to LPC-treated cells (**g**, **h**)

Since oligomeric Aβ promoted MBP synthesis in organotypic cerebellar slices, we next asked the role of Aβ in remyelination after LPC-induced demyelination. First, LPC model was characterized to assess MBP expression recovery after LPC insult in the ex vivo model. As previously described, we observed a sustained MBP level decrease 1 day in vitro (1DIV) after LPC removal and a recovery 6 days in vitro (6DIV) after LPC insult (Fig. 7a–c)^30^. Slices were exposed to Aβ after LPC exposure and MBP levels were analyzed 1 and 6 days after Aβ application (Fig. 7a). Slices treated with Aβ showed a significant and rapid increase in MBP expression relative to controls at same time points (Fig. 7d–f). Notably, Aβ induced an increase in the number of remyelinated fibers after 6DIV of LPC insult (Fig. 7g, h). Moreover, enhancement of MBP expression triggered by Aβ was blocked by PP2 inhibitor (Fig. 7d, e), indicating that Fyn activation led by Aβ oligomers accelerated remyelination after LPC insult in cerebellar slices.

**Fig. 8:**
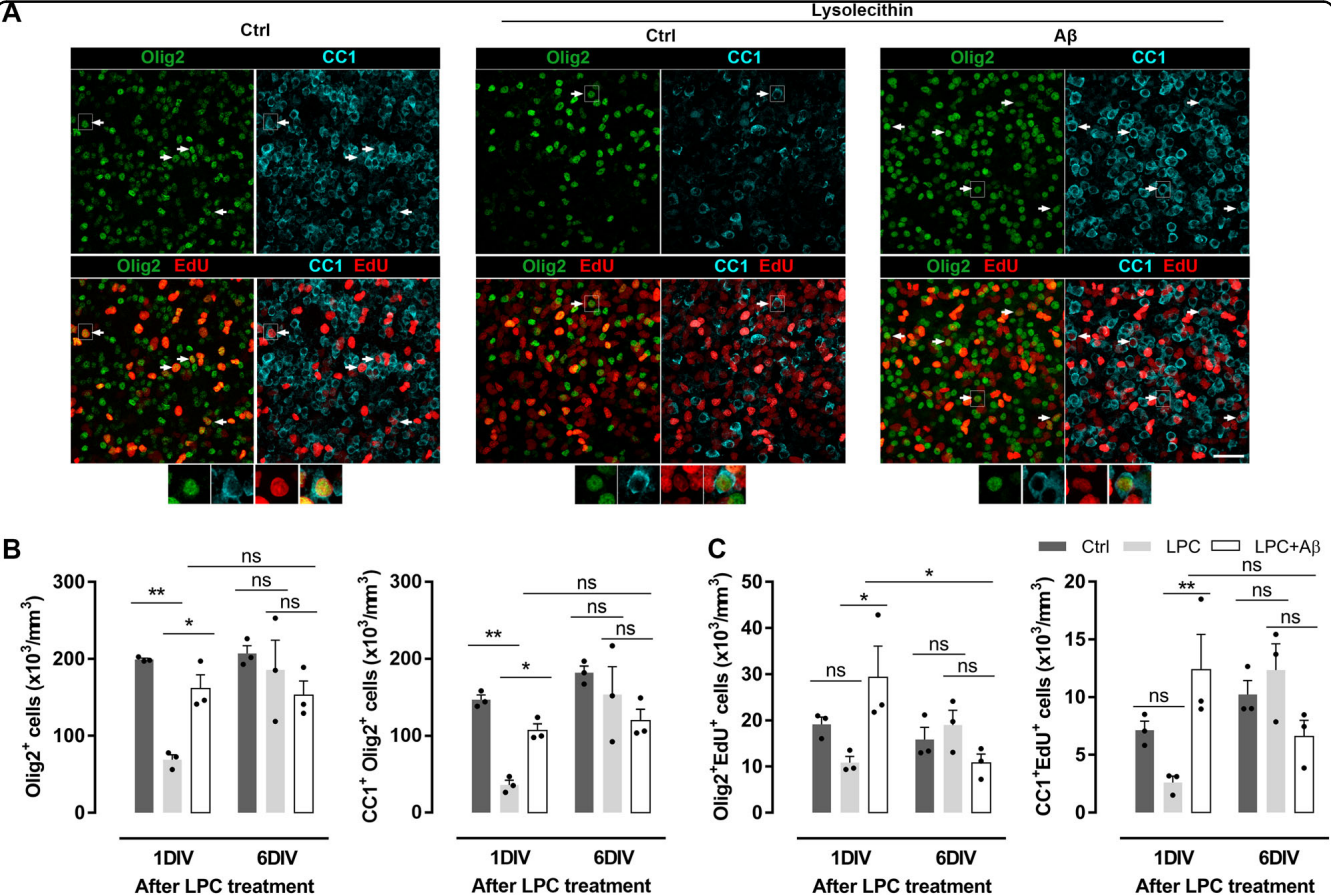
Aβ oligomers promote oligodendrocyte differentiation after LPC demyelination in cerebellar organotypic slices. **a** Staining for Olig2 (green), CC1 (cyan) and EdU (red) in demyelinated cerebellar slices at 1 day post lysolecithin exposure. Scale bar, 50 µm. **b** Mean number of total oligodendrocytes (Olig2^+^) and mature oligodendrocytes (CC1 + Olig2 + ) (*n* = 3). **c** Quantification of new generated Olig2^+^ and CC1^+^ cells after indicated treatments. Arrows indicate new mature oligodendrocytes (CC1^+^Olig2^+^EdU^+^) (*n* = 3). Higher magnifications of new mature oligodendrocytes delimited by a rectangle in **a** (botton panel) are shown. Data are represented as means ± S.E.M and were analyzed by one-way ANOVA followed by Holm-Sidak’s multiple comparisons test. *p < 0.05, **p < 0.01, ***p < 0.001 compared to non-treated slices. #*p* < 0.05 compared to Aβ alone

Next, we quantified the number of oligodendrocyte lineage cells (Olig2^+^) and specifically mature oligodendrocytes (CC1^+^Olig2^+^) after LPC insult. Olig2^+^ and CC1^+^Olig2^+^ cell density was drastically reduced 1DIV after LPC exposure, while slices treated with Aβ showed a robust recovery in oligodendrocyte populations (Fig. 8a, b). However, no differences were found at 6DIV after LPC insult, demonstrating the self recovery capacity of organotypic culture. In addition, we analyzed oligodendrocyte proliferation and differentiation after LPC insult by EdU detection. Aβ promoted the generation of new oligodendrocytes (Olig2^+^EdU^+^) and differentiation to mature cells (CC1^+^EdU^+^) in comparison with LPC-treated slices at 1DIV, while no significant differences were found at 6DIV (Fig. 8a, c). Interestingly, the number of Olig2^+^EdU^+^ cells in Aβ-treated slices was significantly increased 1DIV after LPC insult, but significantly reduced over time (1DIV vs. 6DIV). These results suggest that Aβ restored oligodendrocyte populations and induced their differentiation after demyelination, features, which may contribute to the recovery of the organotypic slices.

## Discussion

Emerging evidence suggest that changes in oligodendrocyte function and myelin may contribute significantly to aging^31^ and brain pathogenesis in AD^32^. To date, however, the molecular events triggered by soluble Aβ oligomers in oligodendrocytes are poorly defined. Our findings indicate that Aβ oligomers coordinated with adhesion protein Itgb1 activate Fyn kinase and CaMKII to promote MBP upregulation, as well as oligodendroglial survival and differentiation in a Ca^2+^-dependent manner. Surprisingly, Aβ oligomers enhance remyelination and oligodendrocyte lineage recovery in LPC-induced demyelination in organotypic slices. Consequently, these data suggest that Aβ oligomers generate dynamic changes on oligodendroglia, and raise an important question about the contribution of Aβ-mediated functions in these cells and in myelin to AD pathogenesis.

MBP protein is defined as an “executive molecule” of myelin^33^ with several multifunctional characteristics. Isoforms of MBP are developmentally regulated and have different cellular distributions. Here, we showed that isoform 18.5 kDa in nominal molecular mass is the mainly expressed in P12 rat optic nerve-derived oligodendrocytes and is specifically modulated by Aβ oligomers. The 18.5 kDa splice variant is produced in large quantities in oligodendrocytes that have been committed to form compact myelin^12^. However, the nuclear 21.5-kDa isoform with effects in oligodendrocyte proliferation^34^ is less expressed in P12 optic nerve cultured oligodendrocytes and its levels are not modified by Aβ. Accordingly, we reported here that Aβ oligomers modulated mechanistically the local translation of 18.5 kDa MBP in oligodendrocyte processes, involving non-receptor tyrosine kinase Fyn activation. MBP mRNA is transported in a translationally silenced state in RNA granules to the myelin compartment at distal processes^35^, where is locally translated by the activation of Fyn kinase at sites of glia-neuron contact^14^. Here, we showed that Fyn was specifically phosphorylated on cultured oligodendrocytes after Aβ treatment, as described in neurons^36^, and the inhibition of Fyn activity by PP2 reduced MBP protein synthesis in cultured oligodendrocytes and in cerebellar slices. Overall, these data suggest that Aβ oligomers regulate the synthesis and compaction of myelin sheets by promoting MBP synthesis through Fyn activation in AD brains.

Fyn kinase is involved in AD pathology^37^. Thus, Aβ-mediated Fyn activation leads to synaptotoxicty in cultured neurons^36^ that in turn, induces synaptic and cognitive impairments in AD models^38,39^, and high levels of Fyn correlate with tau hyperphosphorylation in neurons in AD brain^40^. Thus, Fyn is a potential therapeutic target^37,41^ and its inhibition is currently being evaluated in AD clinical trials^42^. Importantly, we here described that Aβ-mediated Fyn activation in oligodendrocyte triggers changes in their physiology. Specifically, we found that Fyn activity inhibition by PP2 blocked Aβ-induced enhanced oligodendroglial survival and voided oligodendroglial differentiation in organotypic slices. These results confirm a functional link between Aβ oligomers and Fyn kinase mediating differentiation, maturation and survival of oligodendrocytes.

In oligodendrocytes, engagement of integrin receptor family initiates intracellular signaling cascades that are crucial to cell proliferation, survival and maturation^43^. Moreover, previous data have shown that expression pattern of integrins by oligodendrocytes is not only developmentally regulated, but is affected by the nature of surrounding environment^44^. Here, our data showed that Itgb1 mRNA and protein levels were upregulated by Aβ oligomers, similarly to astrocytes^23^, suggesting that Aβ peptides might change the dynamic nature of integrin expression and function in oligodendrocytes throughout AD. Notably, we observed that MBP levels returned to control values when Itgb1 gene expression or activation was reduced by gene silencing or blocking antibodies. These results suggest that Itgb1 participate in the regulation of MBP synthesis by Aβ oligomers, as described previously in normal CNS myelination^45^. Moreover, Aβ-induced Itgb1 expression may result in an increased abundance and availability of molecules of this receptor in the cell surface, exacerbating Aβ-induced MBP upregulation.

MBP levels are influenced by Ca^2+^/CaMKII signaling in Aβ-treated oligodendrocytes. These results are consistent with the effects of Aβ in intracellular Ca^2+^ mobilization described in astrocytes^23^ and in neurons^46,47^. In neurons, Aβ-triggered Ca^2+^ intracellular increment is released from the ER through ryanodine receptors^46,48^. Accordingly, after blocking ryanodine receptors in cultured oligodendrocytes, the increased Ca^2+^ levels triggered by Aβ were attenuated and resulted in a CaMKII decreased activity. Remarkably, inhibition of cytosolic Ca^2+^ release and CaMKII activity resulted in a decrease of MBP, pointing to Ca^2+^ as MBP synthesis mediator and confirming the reported role of CaMKII in regulating oligodendrocyte maturation and myelination^27^.On the other hand, we observed that Aβ promotes an increase in mature oligodendrocytes by inducing cell proliferation and maturation in cerebellar organotypic cultures. Interestingly, application of Aβ to demyelinated organotypic slices resulted in enhanced MBP expression and oligodendrocyte lineage recovery, also supported by newly generated mature cells. The inhibition of Fyn kinase blocked Aβ-induced MBP upregulation. Thus, these data suggest that the stimulation of Fyn by Aβ may initiate a repair process, enhancing oligodendrocyte population and remyelination after demyelinating injury. Collectively, these observations in oligodendrocyte cultures and cerebellar slices show a beneficial effect of Aβ oligomers in oligodendroglial physiology and repair, which adds to those described in vitro and in vivo for memory formation^49,50^.

However, these results are apparently at odds with previous data, which described a toxic effect of Aβ peptides in cerebral cortex-derived oligodendrocytes^51^, and in a mouse oligodendrocyte precursor cell line^52^. These reports showed oligodendrocyte cell damage by a chronic incubation of monomeric Αβ peptide at very high concentrations (4 days at 20 µM). The discrepancies between these studies and the currently reported here may lie on the different Aβ peptide molecular forms, used concentrations and exposure time. It is also conceivable that chronic exposure to higher concentrations of Aβ oligomers could also cause oligodendrocyte death and contribute to the onset of the disease. This possibility may be relevant to AD as local concentrations around or distant to plaques differ and could cause oligotoxicity or promote myelin synthesis. Indeed, we observed that MBP is increased in hippocampal tissue extracts of 6 to 18-month-old 3xTg-AD mice, while the number of CC1^+^ cells is increased^52^, suggesting that changes on myelin properties and oligodendrocyte lineage dynamics could participate in AD pathology. Additionally, the involvement of oligodendrocyte dysfunction in cognitive decline^53^ and the presence of myelin aberrations as result from repair mechanisms mediated by OPCs nearby amyloid plaques^54^ have been described in the APPPS1 mouse model. These findings together with those reported here showing that Aβ induces oligodendrocyte differentiation, suggest that the mechanism underlying OPC-mediated repair in AD models could be a direct response of oligodendrocytes to Aβ, and that Aβ oligomers might not directly cause oligodendrocyte death in AD pathology.

Finally, as oligodendrocytes are highly heterogeneous^55^ it is conceivable that Aβ oligomers may differentially impact a subset of oligodendrocytes. Indeed, we observed that mRNAs of Aβ-signaling mediators are principally expressed in subpopulations described as “newly formed oligodendrocytes” and “myelin-forming oligodendrocytes”, suggesting that both subpopulations might be the main targets for Aβ oligomers. Further studies using specific markers, when available, should be performed to confirm these idea.

In summary, we provide evidence showing that Aβ may promote proliferation, differentiation and survival in oligodendroglia lineage, as well as remyelination after lesion. However, the apparent and intriguing beneficial effects of Aβ may be regional and depending on Aβ oligomer availability and/or concentrations, and do not exclude deleterious effects of Aβ that could eventually trigger oligodendrocyte and myelin breakdown around plaques and other brain areas in AD.

## Materials and methods

### Animals

All experiments were conducted under the supervision and with the approval of our internal animal ethics committee (University of the Basque Country, UPV/EHU). Animals were handled in accordance with the European Communities Council Directive. All possible efforts were made to minimize animal suffering and the number of animals used.

### Preparation of amyloid β-peptides

Oligomeric and fibrillar amyloid-β (Aβ_1–42_) was prepared as reported previously^19,56^. Briefly, Aβ_1–42_ (Bachem, Germany) was initially dissolved in hexafluoroisopropanol (HFIP, Sigma, St. Louis, MO, USA) to a concentration of 1 mM. For the aggregation protocol, the peptide was resuspended in dry dimethylsulfoxide (5 mM; Sigma, St. Louis, MO, USA). Hams F-12 (PromoCell, LabClinics, Barcelona, Spain) or HCl (10 mM) was added to adjust the final peptide concentration to 100 μM to obtain oligomers (4 °C for 24 h) or fibrils (37 °C for 24 h), respectively. Monomeric Aβ was dissolved in PBS to a concentration of 100 μM. In all experiments Aβ refers to oligomeric amyloid-β (Aβ_1–42_) unless otherwise stated.

### Oligodendrocyte cultures

Primary cultures of oligodendrocytes derived from optic nerves of 12-days-old Sprague Dawley rats were obtained as described previously^57^. Cells were seeded into 24-well plates bearing 14-mm-diameter coverslips coated with poly-d-lysine (10 mg/ml) at a density of 5 × 10^3^ cells per well. Cells were maintained at 37 °C and 5% CO_2_ in a chemically defined medium^57^. For the analysis of galactocerebroside/5-Ethynyl-2´deoxyuridine-positive cells (GalC^+^EdU^+^) cells, T3 and T4 hormones were removed from cultured medium. After 1–2 days in vitro, cultures were composed of at least 98% O4^+^GalC^+^ cells; the majority of the remaining cells were stained with antibodies to glial fibrillary acidic protein (GFAP). Microglial cells were not detected in these cultures^58^.

### Cerebellar organotypic culture

Organotypic cerebellar slice culture were prepared from cerebellar sections of P5‐P7 or P12 Sprague Dawley rat pups according to previously described procedures^59,60^. Briefly, cerebella were cut into 350 μm parasagittal slices using a tissue chopper (McIlwain) and meninges were removed. Slices were transferred to 0.4 μm culture membranes inserts (Millipore), each containing three slices. Slices were maintained in six‐well plates in 50% basal medium with Earle´s salt (Life Technologies), 25% Hank´s buffered salt solution (Life Technologies), 25% inactivated horse serum (Life technologies), 5 mg/ml glucose (Panreac), 0,25 mM l‐glutamine (Sigma‐Aldrich) and Antibiotic‐Antimycotic solution (100 units/ml of penicillin, 100 μg/ml of streptomycin and 25 μg/ml of amphotericin B, Life Technologies) at 37 °C in a humidified atmosphere with 5% CO2. Slices were kept in culture for 7 days before performing the experiments.

Lysolecithin (LPC)‐induced demyelination experiments were performed in cerebellar organotypic slices from P12 rats^30^. Slices were cultured 7 days in vitro and treated 15–16 h with LPC 0.5 mg/ml. After that, slices were exposed to Aβ 200 nM for 48 h and were fixed or proteins were extracted 1 and 6 days after Aβ treatment exposure. PP2 inhibitor (10 μM, Selleckchem) was added 30 min before Aβ insult.

### Western blot

Oligodendrocytes and cerebellar slices were exposed to Aβ peptides as is indicated. Antagonists or inhibitors were added 15 min before treatment. Cells were scraped in sodium dodecyl sulfate sample buffer and cerebellar slices were directly resuspended in the same buffer. All these preparations were performed on ice to enhance the lysis process and avoid protein degradation. Samples were boiled at 95 °C for 10 min, size-separated by sodium dodecyl sulfate polyacrylamide gel electrophoresis (SDS‐PAGE) in 4–20% Criterion TGX Precast gels and transferred to Trans-Blot Turbo Midi Nitrocellulosa Transfer Packs (Bio-Rad, Hercules, USA). Membranes were blocked in 5% BSA in Tris‐buffered saline/0.05% Tween‐20 (TBS‐T) and proteins detected by specific primary antibodies against MBP (#SMI 99, 1:1000; Biolegend), Src [pY418] (pSFK; #44660, 1:500; Invitrogen), Fyn (#sc-16, 1:500; Santa Cruz), Lck (#610097, 1:500; BD Bioscience), Src (#2110, 1:1000; Cell Signaling), Src (total Src, #36D10, 1:1000; Cell Signaling), phosphotyrosine (#T1325, Sigma Aldrich), integrin β1 (#4706, 1:1000; Cell Signaling), phospho‐CREB [Ser133] and CREB (#87G3, #D76D11, 1:1000; Cell Signaling), CNPase ((#C5922, 1:500; Sigma Aldrich), PDGFR‐α ((#sc-338, 1:200; Santa Cruz), GAPDH (#MAB374, 1:2000; Millipore) and β‐actin (#A20066, 1:5000; Sigma‐Aldrich). Membranes were incubated with horseradish peroxidase-conjugated secondary antibodies (1:10000, Cell Signalling Technology, Beverly, MA, USA) and were developed using an enhanced chemiluminescence detection kit according to the manufacturer’s instructions (SuperSignal West Dura or Femto, Pierce). The protein bands were detected with a ChemiDoc XRS Imaging System (Bio-Rad), and quantified by volumen using Quantity One (Bio-Rad) software.

### Immunoprecipitation

Fifty microliters of protein A‐sepharose beads (Abcam) were incubated with 1 μg of specific antibody for 4 h at 4 °C and pelleted antibody‐beads complex was kept. Cells treated with Aβ at 200 nM for 15 min were scraped in RIPA buffer supplemented with protease and phosphatase inhibitor cocktail (Thermo Fisher Scientific) and incubated with the antibody‐beads complex overnight at 4 °C under rotary agitation. The lysate‐antibody‐beads complex was centrifuged and washed with RIPA buffer for three times followed by other centrifugation to obtain the immunocomplex. Finally, protein elution was carried out in 2x sample buffer after boiling the sample at 95 °C for 5 min and centrifugated at 12,000 × *g* for 1 min. Proteins were analyzed by western blot.

### Gene silencing by lentivirus infection

After 4 h of cell seeding, oligodendrocytes were transduced with lentivirus containing non‐targeting or Itgb1‐targeting shRNA (12 μl/ml; Santa Cruz Biotechnology) for 24 h in SATO culture media. On the next day, cells were selected by puromycin (1 μg/ml) for 12 h and were treated in fresh media with Aβ at 1 μM for 24 h and subsequently protein extraction was carried out.

### Calcium imaging

Oligodendrocytes were preincubated with fura‐2 AM (Invitrogen) at 5 μM in culture medium for 30 min at 37 °C and washed as previously described^18^. Experiments were performed in a coverslip chamber, mounted on the stage of a inverted epifluorescence microscope (Zeiss Axiovert 35) equipped with a 150 W xenon lamp Polychrome IV (T.I.L.L. Photonics) and a Plan Neofluar 40x oil‐immersion objective (Zeiss). Cells were visualized with a high‐resolution digital black/white CCD camera (ORCA C4742‐80‐12 AG; Hamamatsu Photonics Iberica). Antagonists were continuously perfused for 30 min before and during the Aβ stimulus. Calcium levels were estimated by the 340/380 ratio method and data were analyzed with Excel (Microsoft) and Prism software (GraphPad Software).

### Reverse transcription PCR

Total RNA was extracted from cultured oligodendrocytes using PureLink RNA Mini Kit (Ambion, Thermo Fisher Scientific, MA, USA). Strand complementary DNA synthesis was carried out with reverse transcriptase Superscript TMIII (Invitrogen, Barcelona, Spain) using random primers. Specific primers for Itgb1, Itgb3, Itgb5, Itgb8, Itga5, and Itga6 were obtained from Qiagen. Real-time quantitative PCR reactions were carried out with 25 ng of reverse transcribed RNA and 300 nM of primers diluted in SsoFast Evagreen Supermix reagent (Bio-Rad, Barcelona, Spain). PCR product specificity was checked by melting curves. Data were normalized to a normalization factor obtained in geNorm Software through the analysis of the expression of four housekeeping genes.

### mRNA fluorescent in situ hybridization (FISH)

After Aβ exposure (1 μM, 24 h) cultured oligodendrocytes were fixed in 4% PFA for 10 min and detection of MBP mRNA was carried out using the QuantiGene ViewRNA ISH Cell Assay (Affymetrix Panomics) according to manufacturer’s instructions. FISH assay was performed in the presence and absence of protease treatment. Background fluorescence was established with a negative probe (BACILLUS S. dapB dihydopicolinate reductase L38424, VF1‐11712) and MBP detection was performed using a specific MBP‐targeting probe (NM_017026 VC1‐15251), both purchased from Affymetrix Panomics. Following in situ hybridization, non protease treated‐cells were blocked with 3 mg/ml BSA, 100 mM glycine and 0.25% Triton X‐100 in 0.1 M PBS and incubated with a antibody against MBP overnignt at 4 °C. After several washes cells were incubated with secondary antibody for 1 h and and mounted with Fluoromount‐G. Cell nuclei was visualized by using DAPI.

### Cell viability assay

Cultured oligodendrocyte were incubated with Calcein-AM (Life Technologies) at 1 µM and 37 °C for 30 min in fresh culture medium and then, were washed in pre-warmed 0.1 M PBS for three times. Emitted fluorescence was measured by a Synergy HT (Biotek) spectrophotometer using excitation wavelength at 485 nm and emission at 528 nm.

### EdU labeling

EdU labeling was performed using Click-iT Alexa Fluor 647 Imaging Kit according to the manufacturer’s instructions (Invitrogen) before immunofluorescence. EdU was added to isolated oligodendrocytes (5 µM) and organotypic slices (10 µM) during Aβ treatment.

### Immunochemistry

Cells and cerebellar slices were fixed in 4% paraformaldehyde for 10 and 40 min, respectively. Samples were washed with PBS, permeabilized and blocked in 4% normal goat serum, 0.1% Triton X‐100 in PBS (blocking buffer) for 1 h and incubated overnight at 4 °C with primary antibodies against MBP (#SMI 99, 1:1000; Biolegend), rat anti‐O4 (1:50, kindly supplied by Dr. Chistine Thompson, University of Glasgow), GalC (#MAB342, 5 µg/ml; Millipore), neurofilament light polipeptide (NFL, #C28E10, 1:200; Cell Signalling), Olig2 (#MABN50, 1:1000; Millipore) and APC (CC1, #OP80, 1:200, Millipore). Samples were washed in PBS and incubated with fluorophore-conjugated Alexa secondary antibodies (1:200) in blocking buffer for 1 h at room temperatue (RT). Cell nuclei were detected by incubation with DAPI (4 μg/ml, Sigma‐Aldrich). Samples were mounted with Fluoromount‐G.

### Image acquisition and analysis

Images were acquired using Zeiss AxioVision microscope or Leica TCS STED SP8 confocal microscope. The settings were kept the same for all samples within one experimental group. Image acquisition was determined automatically on a random field. O4+, GalC+, MBP+, or GalC^+^EdU^+^ cells were counted from at least 13 fields per coverslip using 20x objective. Morphology of MBP^+^ cells was analyzed by using Sholl analysis plugin (starting radius 10 µm, ending radius 150 µm, step size 5 µm) and by measuring MBP occupied area in at least ten fields per condition. All analyses were determined with the Image J software. For FISH analysis, protease assay images were quantified using the Radial Profile Angle plugin of Image J software and values of normalized integrated intensity were obtained. Images acquired from experiments carried without protease, Image J macro was designed and used to automate the analysis. MBP labeling allowed us to select the cell occupied area. Concentric circles at 10 µm intervals emerging from the center of the cell nucleus were generated and MBP mRNA and protein fluorescence signals were quantified after background subtraction. The summed mean pixel intensity per area was calculated from the center of the nucleus to 20-µm radius (nuclear area) and from 20 µm to the end of the cell, as periphery. Cells from organotypic slices were counted blindly along the *z*-stack using a 20x objective in Leica TCS SP8 confocal microscope. At least three different fields from two slices per animal were counted by using LAS AF Lite software (Leica). All images shown are projections from *z*-stacks.

### Statistitical analysis

All data were expressed as mean ± S.E.M. Statistical analysis were performed using absolute values and GraphPad Prism software (GraphPad Software). Comparisons between two groups were analyzed using paired Student’s one-tailed *t*-test or two-way analysis of variance (ANOVA) followed by Bonferroni post hoc test when experiment was performed over time. Comparisons among multiple groups were analyzed by one‐way ANOVA followed by Holm-Sidak’s multiple comparisons test. Statistical significance was considered at *p* < 0.05.

## Supplementary information

Supplementary Information
