# Supplementary material for: Aβ oligomers promote oligodendrocyte differentiation and maturation via integrin β1 and Fyn kinase signaling
Source: Cell Death Dis. 2019 Jun 6;10(6):445. doi: 10.1038/s41419-019-1636-8 (PMC6554322; doi:10.1038/s41419-019-1636-8)
Supplement: Supplementary file 1 — Supplementary Information [file 41419_2019_1636_MOESM1_ESM.docx]

**Supplementary Information**

**FIGURES AND LEGENDS**

**
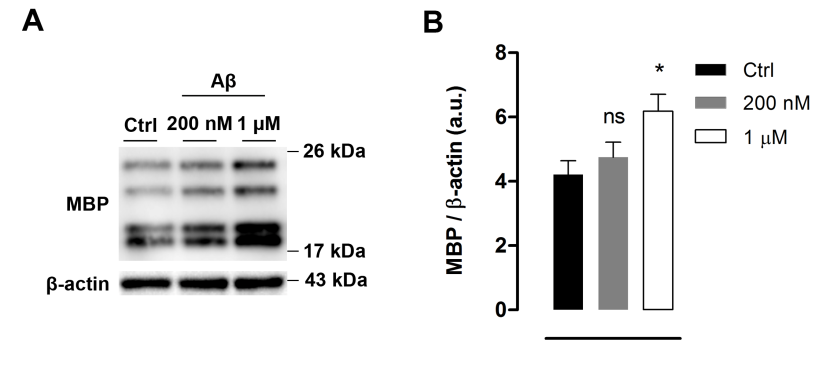
**

**Supplementary Figure 1**. Aβ oligomers modulate MBP expression in cerebral cortex-derived oligodendrocytes. **a, b** Western blotting analysis of the expression of MBP isoforms (21.5 kDa, 18.5 kDa and 17 kDa) and relative quantification in total cell extracts from oligodendrocytes derived from mixed glial cultures obtained from newborn (P0–P2) Sprague–Dawley rat forebrain cortices. Cells were treated with Aβ 200 nM or 1 µM for 24h (n = 5). Data are represented as means ± S.E.M and were analyzed by one-way ANOVA followed by Holm-Sidak’s multiple comparisons test. *p<0.05 compared to non-treated cells.

**
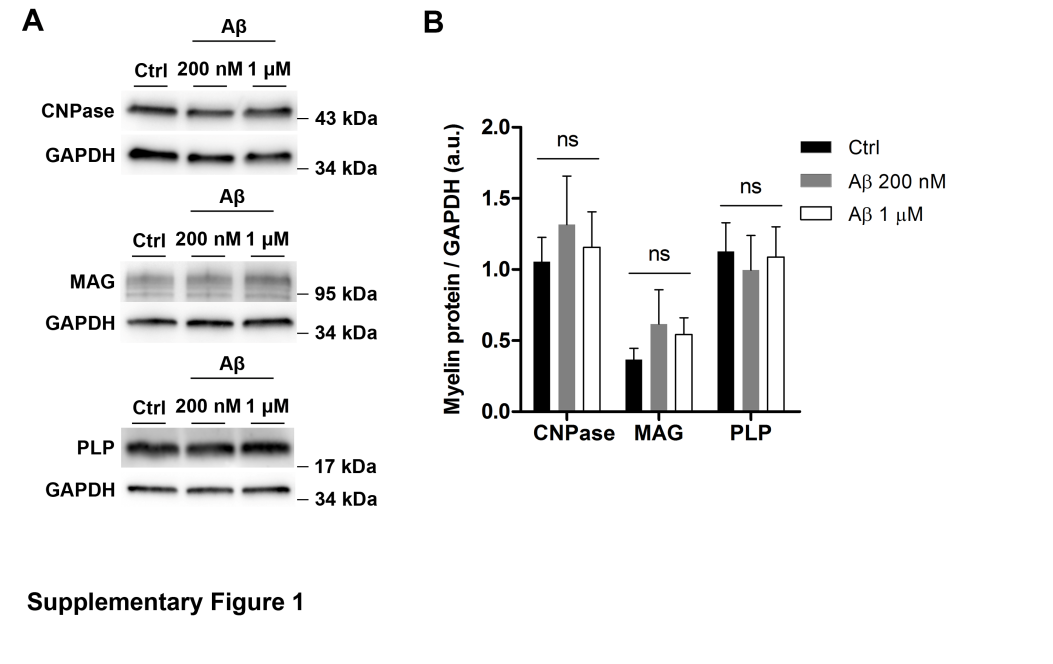
**

**Supplementary Figure 2.** Aβ oligomers do not modulate CNPase, MAG and PLP proteins in cultured oligodendrocytes. **a, b** Western blotting analysis of the expression of CNPase, MAG and PLP and relative quantification in total cell extracts from oligodendrocytes treated with Aβ 200 nM or 1 µM for 24 h was analyzed by western blotting (n = 4). Data are represented as means ± S.E.M and were analyzed by one-way ANOVA followed by Holm-Sidak’s multiple comparisons test.

**
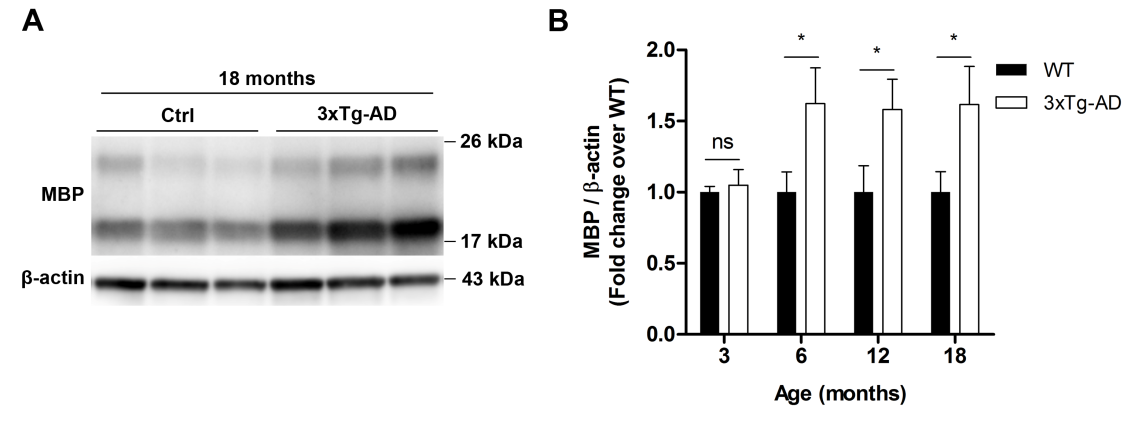
**

**Supplementary Figure 3.** MBP expression is upregulated in AD transgenic mice hippocampus. **a** Western blot of MBP expression in hippocampus of 18‐month‐old 3xTg‐AD mice compared to WT. **b** Quantification of MBP levels in hippocampus of 3-, 6‐, 12‐ and 18‐month‐old 3xTg‐AD mice in comparison with WT (n = 5‐6). Data are represented as means ± S.E.M and were analyzed by unpaired Student´s t test. *p<0.05 compared to WT.


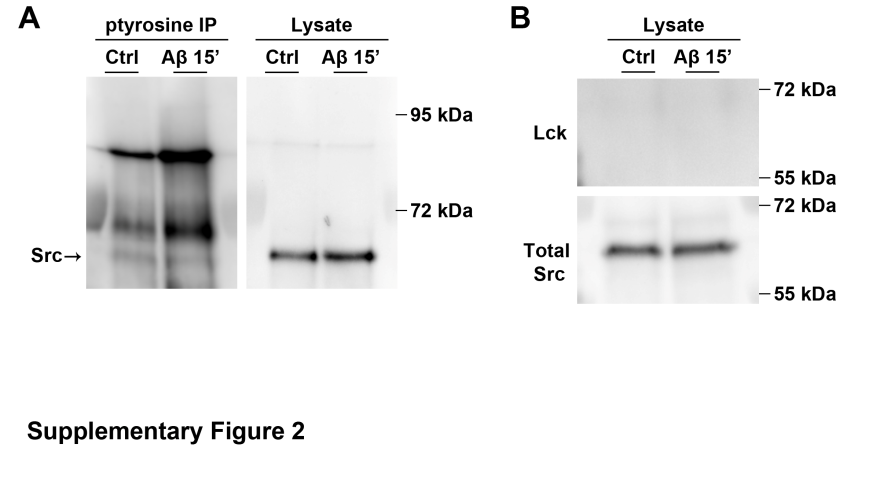


**Supplementary Figure 4.** Aβ oligomers do not promote Src phosphorylation. **a** Immunoprecipitation (IP) of proteins from cells treated with Aβ 200 nM for 15 min was performed with phospho-tyrosine (ptyrosine) followed by Src detection by western blot. Total cell lysates were immunoblotted with Src. **b** Western blot of Lck and total Src in cells treated with Aβ 200 nM for 15 min.

**MATERIAL AND METHODS**

**Oligodendrocyte Culture**

Highly enriched OPCs were prepared from mixed glial cultures obtained from newborn (P0–P2) Sprague–Dawley rat forebrain cortices as previously described (McCarthy and de Vellis, 1980) with minor modifications. Briefly, forebrains were removed from the skulls and the cortices isolated and enzymatically digested by incubation with 0.25% trypsin and 4% DNAse for 15 min at 37°C. Then, the tissue was mechanically dissociated and plated in Iscove’s Modified Dulbecco’s Medium (IMDM) supplemented with 10% fetal bovine serum (FBS, Hyclone). The mixed glial cells were grown in T75 flasks (pre-treated with poly-d-lysine) until they were confluent (10–12 days). Microglia were separated from the cultures by shaking the flasks on a rotary shaker for 1 h at 250 revolutions/min. OPCs were isolated following an additional 18 h. OPCs were seeded on to poly-d-lysine-coated coverslips and were maintained at 37°C and 5% CO_2_ for 2 days in a chemically defined maturation SATO (Canelo-Antelo M *et al*, 2018).

**Protein preparation from 3xTg-AD mice**

Triple transgenic mice of Alzheimer´s disease (3xTg‐AD), which harbours the Swedish mutation in the human amyloid precursor protein (APPSwe), presenilin knock‐in mutation (PS1M146V), and tau P301L mutant transgene (tauP301L) (Oddo *et al*., 2003) were anesthetized with isofluorane (Schering‐Plough) and hippocampi were extracted, placed on dry ice and stored at ‐80ºC. Animal tissue samples were resuspended in 200 μl of RIPA buffer (50 mM Tris pH 7.5, 150 mM NaCl, 0.5% sodium deoxycholate, 0.1% SDS, 1% NP‐40 in 0.1 M PBS) supplemented with protease inhibitor cocktails (Roche), and were homogenized with a glass douncer. Afterwards, they were sonicated for 25 cycles at 80% amplitude (Labsonic M, Sartorius), centrifuged for 10 min, at 1,200 rpm, 4°C, and then supernatants were collected. Total protein content was quantified through Bradford assay (Bio‐Rad). Protein extracts from tissues (10 μg per sample) were analyzed by SDS-PAGE and western blot.

**REFERENCES**

Canelo-Antelo *et al.,* (2018) Inhibition of Casein Kinase 2 Protects Oligodendrocytes From Excitotoxicity by Attenuating JNK/p53 Signaling Cascade**.** Front Mol Neurosci 11:333.

McCarthy KD, de Vellis J (1980). Preparation of separate astroglial and oligodendroglial cell cultures from rat cerebral tissue. J Cell Biol 85:890-902.

Oddo *et al.,* (2003) Triple-transgenic model of Alzheimer's disease with plaques and tangles: intracellular Abeta and synaptic dysfunction. Neuron 39: 409-421.
